# Protein clearance strategies for disease intervention

**DOI:** 10.1007/s00702-021-02431-y

**Published:** 2021-10-23

**Authors:** Franziska Hommen, Saygın Bilican, David Vilchez

**Affiliations:** 1 Cologne Excellence Cluster for Cellular Stress Responses in Aging-Associated Diseases (CECAD), University of Cologne, Joseph Stelzmann Strasse 26, 50931 Cologne, Germany; 2 Center for Molecular Medicine Cologne (CMMC), University of Cologne, Cologne, Germany; 3 Faculty of Medicine, University Hospital Cologne, Cologne, Germany

**Keywords:** Proteostasis, Proteasome, Autophagy, Alzheimer’s disease, Huntington’s disease, Parkinson’s disease, Amyotrophic lateral sclerosis, Aging, Cancer

## Abstract

Protein homeostasis, or proteostasis, is essential for cell function and viability. Unwanted, damaged, misfolded and aggregated proteins are degraded by the ubiquitin–proteasome system (UPS) and the autophagy-lysosome pathway. Growing evidence indicates that alterations in these major proteolytic mechanisms lead to a demise in proteostasis, contributing to the onset and development of distinct diseases. Indeed, dysregulation of the UPS or autophagy is linked to several neurodegenerative, infectious and inflammatory disorders as well as cancer. Thus, modulation of protein clearance pathways is a promising approach for therapeutics. In this review, we discuss recent findings and open questions on how targeting proteolytic mechanisms could be applied for disease intervention.

## Introduction

**Fig. 1:**
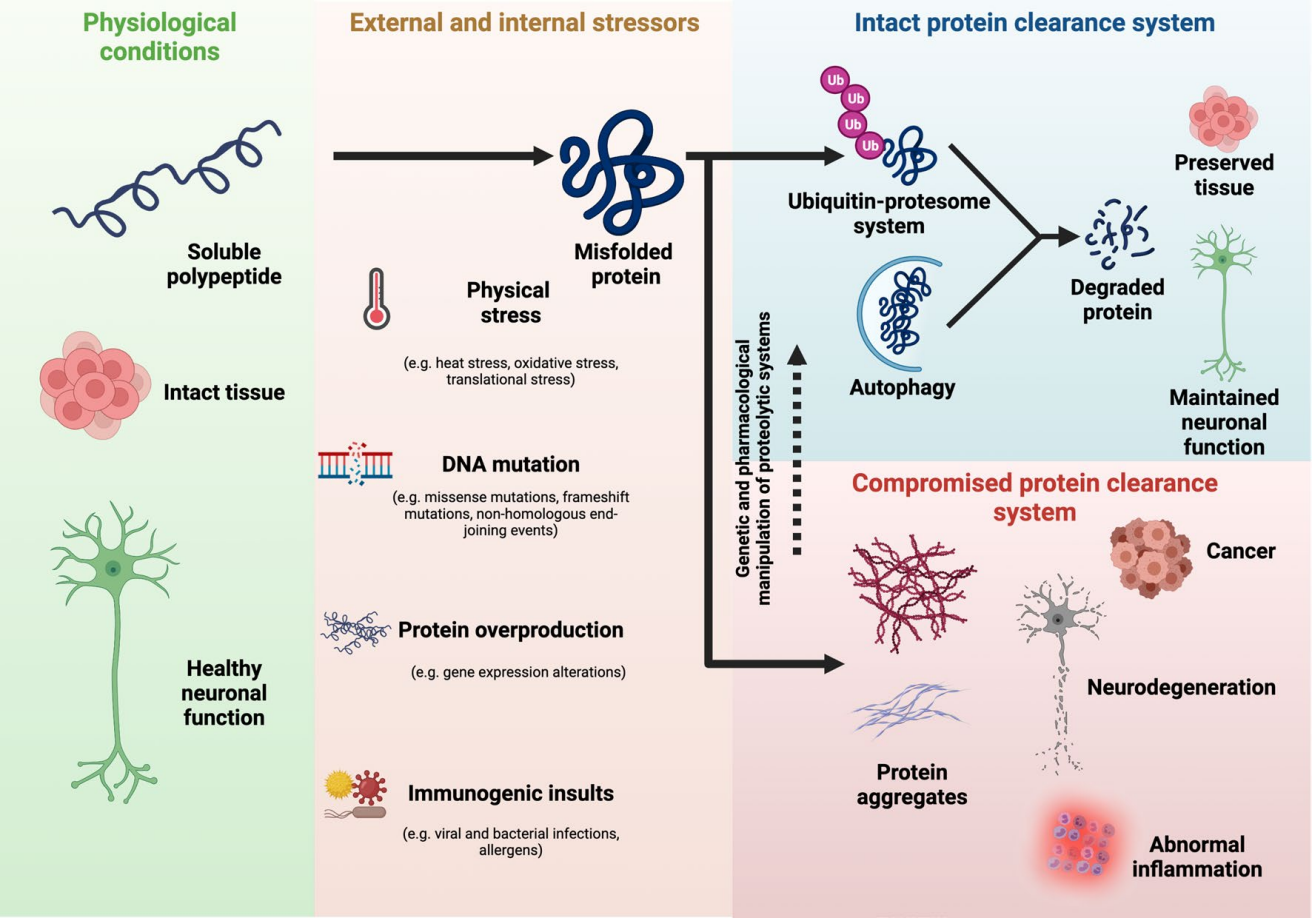
Protein clearance mechanisms in health and disease. Misfolded proteins that ensue from external and internal stressors are degraded through two major protein clearance pathways, i.e., the ubiquitin–proteasome system (UPS) and the autophagy-lysosome pathway. Dysfunction of these pathways contribute to the accumulation of protein aggregates, a hallmark of disorders such as Alzheimer’s disease, Huntington’s disease, Parkinson’s disease and amyotrophic lateral sclerosis

The proteome of a mammalian cell contains thousands of distinct proteins (Jayaraj et al. 2020). The intracellular levels of individual proteins are adjusted to the particular needs and status of every single cell in the organism (Jayaraj et al. 2020). Moreover, numerous proteins are prone to misfolding and aggregation, leading to cell malfunction and death. Thus, maintenance of protein homeostasis (proteostasis) is essential for cell function and survival (Fig. 1). As such, cellular integrity relies on proteolytic systems that not only maintain the proper concentration of regulatory and structural proteins, but also scavenge damaged and misfolded proteins (Saez and Vilchez, 2014; Sha et al. 2018; Vilchez et al. 2014). The evolutionary conserved ubiquitin–proteasome system (UPS) and autophagy-lysosome pathway are the two major protein clearance mechanisms in eukaryotes. However, conditions such as cellular stress, metabolic alterations, pathogens, environmental changes, disease-related mutations and aging can influence proteolytic systems (Hipp et al. 2019; Vilchez et al. 2014).

Deficits in protein folding and clearance mechanisms are linked to multiple disorders that involve protein aggregation, i.e., proteinopathies. The link is more compelling in neurodegenerative disorders such as Alzheimer’s, Huntington’s, Parkinson’s and amyotrophic lateral sclerosis (ALS). Although the proteins involved in these diseases are different, they have in common the accumulation of pathological protein inclusions in neurons. In addition, these neurodegenerative diseases share similar late temporal emergence patterns. Typically, familial mutation-linked neurodegeneration emerges during the fifth decade of life, whereas the onset of sporadic neurodegenerative disease usually occurs during the seventh decade or even later (Cohen and Dillin 2008). For instance, ALS is rare before the age of 40 years, but its incidence increases exponentially thereafter with a peak at 70–79 years of age (Ingre et al. 2015). With age, post-mitotic cells such as neurons lose extensive control of the proteostasis equilibrium, including deficits in protein degradation machineries (Vilchez et al. 2014). Loss of proteostasis is a hallmark of aging, further strengthening a role of proteolytic deficits in the onset of neurodegenerative diseases (Lopez-Otin et al. 2013; Vilchez et al. 2014). In addition, patients with inflammatory and infectious diseases as well as cancer also present changes in proteolytic systems (Dang et al. 2021; Li et al. 2016; Wang et al. 2021a, b). Current research efforts are focused on understanding how alterations in proteolytic systems can contribute to the onset and prognosis of disease with the aim to identify novel therapeutic approaches (Wang et al. 2021a, b). Here we review recent discoveries and how they may develop into promising therapies for proteinopathies.

## The ubiquitin–proteasome system (UPS)

**Fig. 2:**
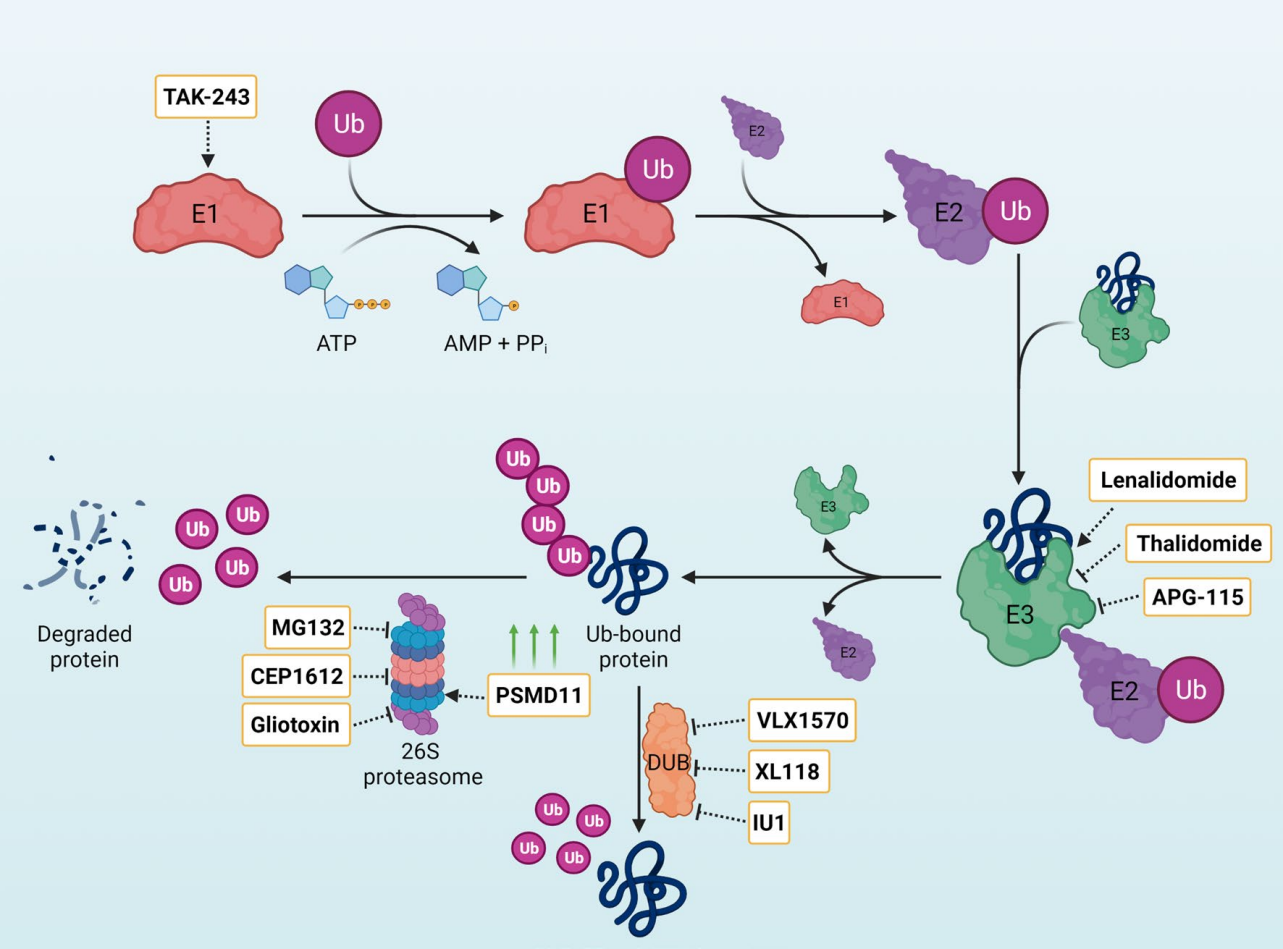
Modulation of the ubiquitin proteasome system (UPS) for disease intervention. Ubiquitin (Ub) binds to the Ub-activating enzyme E1 by a thioester bond in an ATP-dependent manner and then is transferred to the E2 enzyme. The attachment of Ub to the target protein is catalysed by E3 ligases. This process can be reversed by deubiquitinating enzymes (DUBs). Ub-tagged proteins are recognized and degraded by the 26S proteasome. Inhibitors and activators of the UPS are indicated with dashed lines

The UPS is the primary selective proteolytic system in mammalian cells, regulating numerous biological processes such as development, gene transcription, signal transduction, metabolism, apoptosis, cell cycle, DNA repair and inflammation (Chen et al. 2008; Melino 2005; Wang and Maldonado 2006; Yao and Ndoja 2012). In the UPS, lysine residues of proteins are tagged with the small protein ubiquitin (Ub) to enable their recognition by the proteasome (Hershko and Ciechanover 1998) (Fig. 2). The covalent attachment of Ub to a substrate protein is catalyzed by a sequential cascade of three enzymatic reactions, starting with Ub activation by the Ub-activating enzyme E1 (UBA1) in an ATP-dependent reaction (Lambert-Smith et al. 2020). Adenylation of Gly76 at the C-terminus of Ub is followed by a thioester bond formation between UBA1 and Ub (Lambert-Smith et al. 2020). Thereafter, Ub-conjugating enzymes (E2s) are recruited by the C-terminal ubiquitin-fold domain of UBA1 where Ub is transferred to the E2 enzyme (Hershko and Ciechanover 1998). The resulting thioester intermediate dissociates from UBA1 and, together with the target protein, binds to a specific E3 ubiquitin ligase (Hershko and Ciechanover 1998). Then, E3 ligases catalyze the covalent attachment of Ub to the target protein. In humans, there are more than 600 E3 enzymes which can be distinguished into two main classes, i.e., RING-type and HECT-type E3s (Plechanovová et al. 2012). Whereas E3 ligases mark proteins with Ub, deubiquitinating enzymes (DUBs) can reverse this process (Wilkinson 2000). To date, 102 DUBs have been reported in humans (Clague et al. 2019; Pinto-Fernández et al. 2019). Thus, the activities of E3 ligases and DUBs are tightly balanced to maintain intracellular proteostasis and cellular function (Bax et al. 2019; Choi and Baek 2018). In these lines, the landscape of E3 and DUB enzymes undergo a profound rewiring during transformative processes such as cell differentiation or organismal aging (Koyuncu et al. 2021; Saez et al. 2018).

Target proteins can be tagged with Ub at one lysine residue or multiple lysine residues. Moreover, Ub itself harbors seven internal lysine residues that can form polyUb chains. A Lys48-linked polyUb chain is the primary signal for recognition and degradation by the 26S proteasome, a multi-catalytic/multi-subunit protease complex that degrades polyubiquitinated proteins to small polypeptides (Hershko and Ciechanover 1998). In addition to lys48, other Ub linkages such as Lys63 or heterotypic chains can also target proteins for degradation (Yau et al. 2017).

## Autophagy

**Fig. 3:**
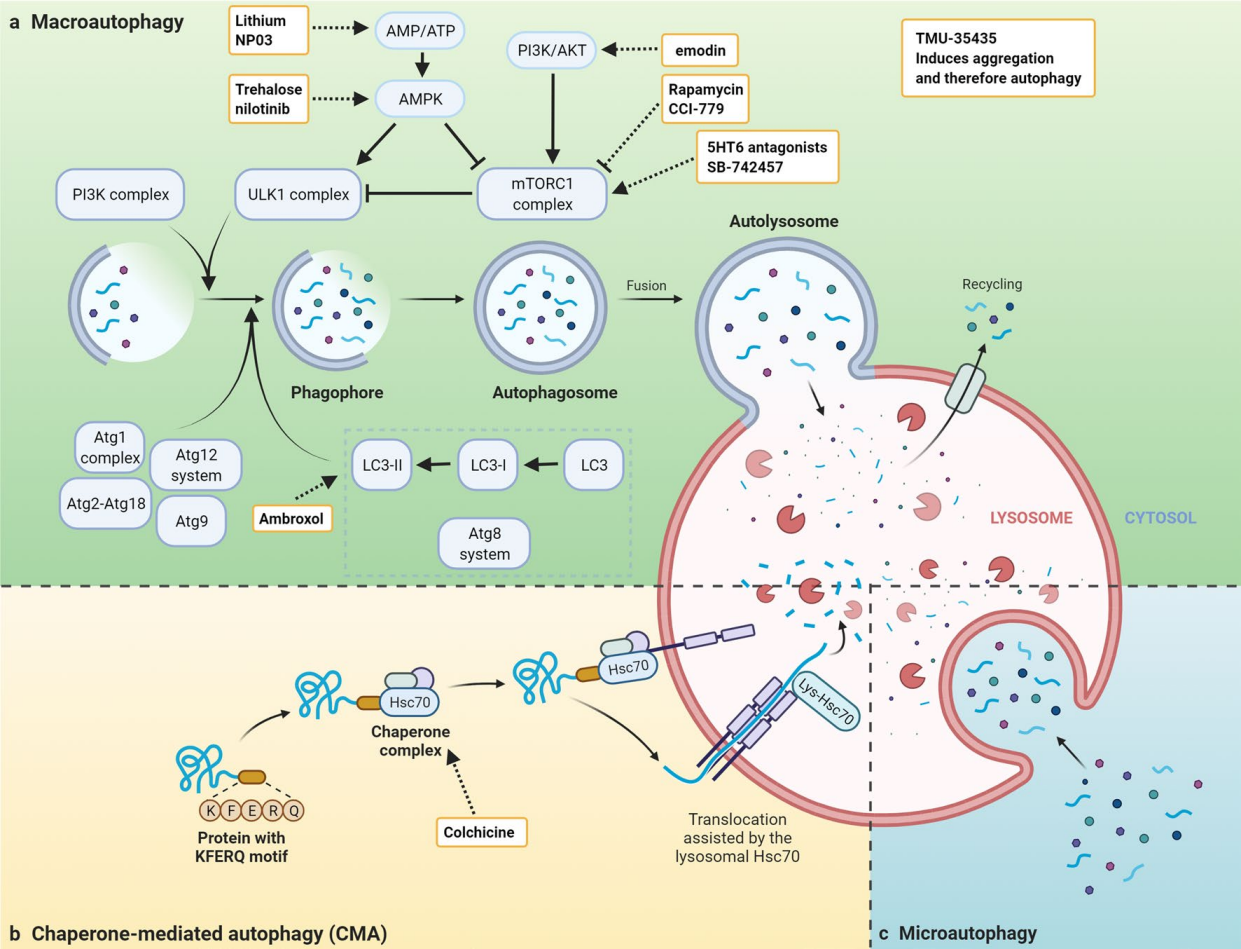
Modulation of autophagy for disease intervention. **a** Schematic overview of the macroautophagy pathway. Macroautophagy is induced by inhibition of mTORC1 complex and starts with the formation of a phagophore which matures into the autophagosome. The autophagosome fuses with the lysosome to transfer its cargo. Most inhibitors and activators target the mTORC1 complex either directly or indirectly. **b** Schematic overview of chaperone-mediated autophagy (CMA). Proteins harbouring a KFERQ motif are recognized by Hsc70 and translocated to the lysosomal lumen through interaction with LAMP2A. **c** Schematic overview of microautophagy. Cytosolic substrates are directly transported to the lysosomal lumen. Inhibitors and activators are indicated with dashed lines

The autophagy-lysosome pathway has a central role in biological processes such as cell differentiation, proliferation, and senescence. Autophagy transfers cytosolic substrates to the lysosome for degradation either in a selective or non-selective manner. The autophagy-lysosome pathway can be distinguished into three types, namely macro-, micro-, and chaperone-mediated autophagy (CMA) (Fig. 3a–c) (Klionsky et al. 2011; Levine and Klionsky 2004; Mizushima 2007).

### Macroautophagy

Macroautophagy is the most characterized pathway of autophagy. Macroautophagy not only promotes the recycling of damaged organelles such as mitochondria (mitophagy) and the endoplasmic reticulum (reticulophagy), but also degradation of protein aggregates that cannot be cleared by the UPS (Tasdemir et al. 2007; Youle and Narendra 2011). Macroautophagy is induced by the inactivation of the mammalian target of rapamycin complex 1 (mTORC1), a serine/threonine kinase complex which is sensitive to intra- and extracellular nutrient levels (Ben-Sahra and Manning 2017; Rabanal-Ruiz et al. 2017). Concomitantly, TOR inhibitors such as rapamycin and CCI-779 induce autophagy (Blommaart et al. 1995; Dudkin et al. 2001; Yu et al. 2001). Macroautophagy starts with the formation of a phagophore, a vesicle surrounding cytoplasmic material, which turns into the so-called autophagosome. In yeast, autophagosome formation requires 18 autophagy-related (Atg) proteins. The pre-autophagosomal structure is formed by six functional groups of Atg complexes which are highly conserved among eukaryotes, i.e., the Atg1 autophagy initiation complex, Atg9, Atg2-Atg18 complex, the autophagy-specific phosphatidylinositol 3-kinase (Pi3K) complex, the Atg12-Atg-5 conjugation system, and the Atg8-Atg18 Ub-like conjugation system (Suzuki et al. 2017). The closure of the autophagosome is driven by the endosomal sorting complex required for transport (ESCRT) proteins, with CHMP2A being the main regulator (Takahashi et al. 2018). Then, the autophagosome transfers its cargo such as organelles and protein aggregates to the lysosome. During this process, the outer membrane of the autophagosome fuses with the lysosome which then degrades the inner membrane through acidic hydrolases.

### Microautophagy

Microautophagy plays an essential role in cell survival. In contrast to macroautophagy, which requires the formation of an autophagosome, microautophagy transports cytosolic substrates directly to the lysosomal or endosomal lumen (Marzella et al. 1981). In yeast, some types of microautophagy are driven by ESCRT machinery. Particularly, inhibition of mTORC1 upon starvation leads to dephosphorylation of Vps27, a component of ESCRT-0, resulting in the initiation of microautophagy through ESCRT-0 (Hatakeyama and Virgilio 2019). However, the mechanisms underlying microautophagy in multicellular eukaryotes remain elusive.

### Chaperone-mediated autophagy (CMA)

Degradation of proteins through chaperone-mediated autophagy (CMA) is mediated by the cytosolic heat shock-cognate chaperone of 70 kDa (Hsc70), also known as heat shock 70 kDa protein 8 (HSPA8). HSPA8 recognizes substrates that contain a specific pentapeptide motif (KFERQ) (Chiang et al. 1989; Dice 1988). This pentapeptide motif becomes accessible when the target protein changes its binding properties or conformation, exposing the KFERQ-motif. The resulting complex interacts with the lysosome-associated membrane protein type-2A (LAMP2A). The binding of LAMP2A to a substrate protein leads to a high-weight multi-protein complex which is required for translocation into the lysosomal lumen (Bandyopadhyay et al. 2008). During the assembly of the LAMP2A-protein complex, the target protein is unfolded to enter the lysosomal lumen through a lysosomal membrane receptor/translocation complex, a process mediated by Hsc70 and heat shock protein 90 (Hsp90).

## Diseases triggered by protein misfolding and aggregation

The pathological accumulation and aggregation of misfolded proteins is a phenomenon observed in many disorders, including distinct neurodegenerative diseases. Alzheimer’s disease (AD), the most common cause of dementia, is characterized by the deposition of two different protein aggregates: (a) senile amyloid-β (Aβ) plaques and (b) neurofibrillary tangles of the microtubule-associated protein tau (Nie et al. 2011; Penke et al. 2017; Selkoe, 2011; Snyder et al. 1994). These aggregates can result in synaptic dysfunction and neurodegeneration (Selkoe 2011). Parkinson’s disease (PD), the most common movement disorder with age, is characterized by the aggregation of misfolded α-synuclein (α-syn), leading to inclusions known as Lewy bodies (Arima et al. 1998; Fares et al. 2021; Takeda et al. 1998). Malfunction of the UPS, CMA and lysosomes are observed in early stages of the disease, suggesting that dysregulation of these proteolytic systems is involved in the pathogenesis of PD (Alvarez-Erviti et al. 2010; Leroy et al. 1998; McNaught et al. 2001). Huntington’s Disease (HD) is caused by the expansion of the polyglutamine (polyQ) tract of the huntingtin protein (HTT). Whereas the wild-type HTT protein contains less than 35 polyQ repeats, an expansion of > 35 polyQ repeats can lead to HD (Shannon 2011; Yushchenko et al. 2018). Expanded polyQ-mutant HTT tends to aggregate in different in vitro and in vivo models. Indeed, cumulative evidence indicates that mutant HTT aggregates directly contribute to neurodegeneration phenotype in HD (Brignull et al. 2006; Djajadikerta et al. 2020; Gruber et al. 2018; Koyuncu et al. 2018; Nagai et al. 2000).

Familial cases of ALS are linked with mutations in one of > 25 different genes that act in a variety of cellular processes. A handful of genes harbor the majority of familial ALS mutations, including *SOD1*, *TDP-43, FUS* and *C9orf72* (Bartoletti et al. 2019). Familial ALS-related mutations in TDP-43 and FUS proteins induce their cytosolic aggregation (Bentmann et al. 2013; Liu-Yesucevitz et al. 2010). Importantly, TDP-43 and FUS-immunoreactive aggregates are also a common feature in sporadic ALS (Giordana et al. 2010; Hx et al. 2010). Likewise, cytosolic inclusions of TDP-43 is also a characteristic of frontotemporal dementia (FTD), the most common form of early-onset dementia (Arai et al. 2006; Neumann et al. 2006). Expanded hexanucleotide (GGGGCC) repeats in the first intron of *C9orf72* are the most common cause of familial ALS, accounting for approximately 40% of familial cases (DeJesus-Hernandez et al. 2011; Renton et al. 2011). The hexanucleotide expansions range between 100 and 4000 repeats in patients, generating homopolymeric dipeptide proteins (e.g., poly-GR, GP, GA, PR) which are prone to aggregation (Ash et al. 2013; Mori et al. 2013). In these lines, *C9orf72*‐associated cases exhibit neuropathological changes characterized by abundant protein inclusions (Cooper-Knock et al. 2012), indicating a link with proteostasis deficits.

Besides neurodegenerative diseases, other disorders are also linked with protein misfolding and aggregation. For instance, the protein p53 forms aggregates in several types of cancer. Missense mutations in *TP53*, the gene encoding for p53, have been reported in around 50% of cancerous tumors (Carson and Lois 1995). Protein aggregation is also observed in viral infections. For instance, the herpesviruses murine cytomegalovirus and herpes simplex virus 1 induce the aggregation and degradation of NF-kappa-B essential modulator (NEMO) and receptor-interacting serine/threonine-protein kinase 1 (RIPK1) to block the innate immune response (Muscolino et al. 2020). Furthermore, the microtubule-associated proteins 1A/1B light chain 3B (LC3) adaptor TBC1D5 was identified as an autophagy receptor for virus-induced protein aggregates (Muscolino et al. 2020).

Given the extensive list of diseases characterized by protein aggregates, it is not surprising that many studies focused on how protein clearance mechanisms impinge on disease and their potential for therapeutics. In Table 1, we have summarized reported components of the UPS and autophagy which could be potential targets for disease intervention. The following sections will provide an overview about recent findings and ongoing trials based on protein clearance mechanisms.

**Table 1:** List of components of the UPS and autophagy related to proteinopathies

Gene name	Model	Abnormality/Disease model	Aggregated/Accumulated protein	Mutation	Source
PIK3C3	Zebrafish	Postnatal lethality	E-cadherin	PIK3C3 knockout	Zhao et al. (2018)
PTEN	Mouse, COS-7 cells	AD	tau aggregation	PTEN phosphatase-null mutation	Zhang et al. (2006a,b)
TRAF6	HEK cells	PD	alpha-synuclein, ubiquitinated mutant DJ-1	DJ-1^L166P^ TRAF6 overexpression	Zucchelli et al. (2010)
INS	–	AD	Aβ1-42 (insulin prevents Aβ1-42 aggregation in vitro)	–	Long et al. (2019)
TBK1	Mouse	FTD, ALS	p62	SOD1^G93A^ TBK1^R228H/R228H^	Gerbino et al. (2020)
IRS2	Mouse	AD	Reduction in aggregated Aβ	APP^K670N, M671L^ IRS2 knockout	Killick et al. (2009)
		HD	Reduction in aggregated HTT	R6/2 mice Brain specific, heterozygous IRS2 knockout	Sadagurski et al. (2011)
TSC1	HEK cells	TSC	TSC1	Truncated TSC1	Hoogeveen-Westerveld et al. (2010)
TSC2	HEK cells	TSC	Solubilizing TSC1 aggregates	Truncated TSC1 TSC2 co-expression	Hoogeveen-Westerveld et al. (2010)
RAB39B	Human	PD	Lewy Bodies, alpha-synuclein	Complete deletion of RAB39B	Wilson et al. (2014)
BECN	Mouse, HeLa cells	HIV, Chikunguya and West Nile virus infection, HD	PolyQ in HeLa	Heterozygous deletion of BECN in mouse Expanded polyQ in HeLa	Shoji-Kawata et al. (2013)
		AD	Aβ	Human APP Heterozygous deletion of BECN	Pickford et al. (2008)
		HD	PolyQ	Expanded polyQ in HeLa	Ashkenazi et al. (2017)
ATG7	Mouse	Neurodegeneration	Accumulation of ubiquitinated proteins	Conditional knockout of ATG7	Komatsu et al. (2006)
ATG5	Mouse	Cataracts	Ubiquitin and p62 positive aggregates	Lens specific ATG5 knockout	Morishita et al. (2013)
RB1CC1	Mouse	Neurodegeneration	Ubiquitinated protein	Neural-specific deletion of RB1CC1	Liang et al. (2010)
ATG16L1	Mouse	Crohn's disease like ileitis	IRE1α aggregates	ATG16L1 deletion in intestinal epithelial cells	Tschurtschenthaler et al. (2017)
ATG2	HeLa cells	–	Aggregation of LC3 and lipid droplets	siRNA knockdown of ATG2	Velikkakath et al. (2012)
ATG9	Mouse	axon-specific lesions	Accumulation of ubiquitinated proteins	Conditional knockout of ATG9	Yamaguchi et al. (2018)
AMBRA1	Mouse	Embryonic lethality	Accumulation of ubiquitinated proteins	AMBRA1 gene-trapped in LacZ	Maria Fimia et al. (2007)
MTMR14	Fruit Fly	AD and HD	Knockdown of MTMR14 decreases Aβ1-42 and polyQ aggregates	Knockdown of MTMR14	Xiao et al. (2019)
RUBCN	Worm	HD	Knockdown of RUBCN decreases polyQ aggregates	Knockdown of RUBCN	Nakamura et al. (2019)
ATG101	Fruit Fly	Neurodegeneration	Accumulation of ubiquitinated proteins	Loss-of-function mutation of ATG101	Guo et al. (2019)
VMP1	Mouse	PD	LC3, p62, alpha-synuclein aggregation	Deletion of VMP1 in dopaminergic neurons	Wang et al. (2021a; b)
UVRAG	Mouse	Inflammation and Tumorigenesis	Parkin and p62 positive aggregates	Inducible UVRAG truncation mutant	Quach et al. (2019)
C9ORF72	Mouse, Fruit Fly, Worm	ALS	DPR aggregation, RNA foci	Hexanucleotide repeat expansion, expression of DPR constructs	Jiang et al. (2016), Rudich et al. (2017), Xu and Xu (2018)
PSMD12	Yeast	–	Aggregation of PSMD12	Truncation of PSMD12 in C-terminal	Peters et al. (2015)
PSMD11	Mouse	Embryonic lethality	Accumulation of ubiquitinated proteins	Inducible PSMD11 knockout	Zhao et al. (2021)
PSMC4	Mouse	Muscle atrophy	Ubiquitin positive aggregates	PSMC4 knockout in muscle	Kitajima et al. (2014)
PSMC6	HeLa	HD	PolyQ aggregation	Expanded polyQ in HeLa PSMC6 overexpression	Rousseau et al. (2009)
PSMC3	Human	Cataracts and deafness	Accumulation of ubiquitinated proteins	missense mutation of PSMC3	Kröll-Hermi et al. (2020)
PSMC5	HeLa	HD	PolyQ aggregation	Expanded polyQ in HeLa PSMC5 overexpression	Rousseau et al. (2009)
PSMF1	Mouse	Neurodegeneration, embryonic lethality	p62 aggregates at neuromuscular junction, accumulation of ubiquitinated proteins	Inducible PSMF1 knockout	Minis et al. (2019)

The disease model, accumulated/aggregated proteins and the relevant mutations are indicated in the table. The list of selected genes were obtained from Kyoto Encyclopedia of Genes and Genomes proteasome (map03050) and autophagy- animal (map04140)

## Ubiquitin–proteasome system in disease

The UPS can modulate the levels of dysregulated proteins and terminate aggregation-prone proteins. As such, UPS dysfunction has been linked to multiple disorders. Since proteasomal degradation of target substrates can be modulated at different steps from ubiquitination to proteolytic activity, it provides a mean to prevent proteotoxicity through different pharmacological and genetic approaches (Fig. 2).

### Targeting the UPS in neurodegenerative diseases

Deficiencies in proteasome-mediated degradation contribute to neurodegenerative diseases, including AD, PD, ALS, FTD and HD. For instance, tissue samples from patients exhibit aggregates containing Ub (Lowe et al. 1988; Perry et al. 1987). Parkin is one of the most studied E3 enzymes in the context of neurodegeneration and proteotoxicity. Parkin is a RING-between-RING E3 ligase which, together with the serine/threonine kinase PINK1, has a crucial role in mitochondrial quality control and mitophagy (Beasley et al. 2007; Capili et al. 2004; Fett et al. 2010). Under physiological conditions, the two RING domains of parkin are blocked, leading to its inactivation (Duda et al. 2013; Seirafi et al. 2015). Upon severe mitochondrial damage, the mitochondrial membrane becomes depolarized and recruits parkin. The activation of parkin requires both binding of a phospho-Ub and phosphorylation by PINK1 (Gladkova et al. 2018; Koyano et al. 2014; Wauer et al. 2015). Activated parkin ubiquitinates voltage-dependent anion-selective channel 1 (VDAC1), which is only exposed when mitochondria are depolarized. Subsequently, the autophagy adaptors p62, CALCOCO2 and TAX1BP1 are recruited by parkin to initiate the autophagosome formation (Sarraf et al. 2013). There are more than 120 PD-relevant mutations reported in parkin (Cruts et al. 2012; Seirafi et al. 2015). These mutations lead to loss-of-function (LOF), either by compromising parkin integrity or preventing parkin from recognizing its substrates (Wauer and Komander 2013). Cells derived from patients are a useful resource for understanding the role of parkin in PD. Fibroblasts obtained from a family with familial parkin mutations display reduced ATP synthesis, total ATP levels and membrane potential compared to control fibroblasts, indicating mitochondrial dysfunction (Grünewald et al. 2010). Moreover, there is a global increase in oxidized proteins in parkin mutants, a sign of increased reactive oxygen species (ROS). An in vitro study by Jiang et al. successfully produced dopaminergic neurons from iPSCs derived from PD patient fibroblasts (Jiang et al. 2012). These neurons have spontaneous dopamine release, decreased dopamine intake, and elevated ROS levels.

Besides regulating mitochondrial function, parkin impacts on PD-related neurodegeneration by interacting with α-syn, which is one of the substrates of parkin (Norris et al. 2015). Several studies demonstrated that parkin overexpression can have beneficial effects on α-syn toxicity in vitro and in vivo whereas loss of parkin results in the accumulation of α-syn (Meng et al. 2020; Petrucelli et al. 2002; Rana et al. 2013; Shimura et al. 2001). Transduction of mutant α-syn in the mouse midbrain leads to a sharp decrease in dopaminergic neurons and proteasome activity, which is rescued by parkin induction (Petrucelli et al. 2002). A similar study conducted in fruit flies overexpressing both mutant α-syn and parkin in dopaminergic neurons showed that even though parkin improves the survival of these neurons, the levels of α-syn levels remained similar when compared to flies that did not overexpress parkin (Yang et al. 2003).

The impact of parkin on the clearance of aggregated proteins extends beyond PD models. Aβ plaques, which are a common hallmark of AD, can cause mitochondrial swelling, decreased cristae and impaired mitophagy in HEK293 human cell lines. Importantly, parkin overexpression successfully restores mitophagy and reverses mitochondrial fragmentation in Aβ-treated HEK293 human cell lines (Wang et al. 2020a,b). Moreover, parkin can diminish toxicity and aggregation of ALS-related SOD1 mutant variants in SH-SY5Y neuroblastoma cells. In particular, parkin promotes Lys63-linked polyubiquitination of misfolded SOD1 in cooperation with UbcH13/Uev1a E2 enzyme, triggering the clearance of aggregation-prone SOD1 through autophagy (Yung et al. 2016). In these lines, in vivo studies on parkin also led to promising results. Overexpression of parkin in *D. melanogaster* extends longevity, without affecting reproductivity, organismal activity and food intake (Rana et al. 2013). Parkin-overexpressing flies have more Lys48-linked polyubiquitinated proteins and less protein aggregates compared with their wild-type counterparts. The ameliorative effects of parkin could be partially explained by its role in degradation of mitofusin, a protein that induces mitochondrial fusion and could eventually promote mitochondrial impairment when upregulated (Poole et al. 2010; Rana et al. 2013; Tanaka et al. 2010). In *Drosophila*, the levels of mitofusin protein increase during aging. However, overexpression of parkin reduces mitofusin levels in aging flies, with subsequent changes in mitochondrial morphology and increased mitochondrial activity (Rana et al. 2013). Given the potential beneficial role of parkin as a disease modifier, several studies sought to define parking activators. These findings led to the discovery and patent of US-2016/0160205A1 and WO-2018/023029 as small molecule activators of parkin. Currently, there are no in vitro or in vivo data available regarding these molecules (Clark et al. 2020; Miller and Muqit 2019).

Whereas several findings indicate a protective role of parkin, it is important to note that parkin activity could also have negative effects depending on the disease model. For instance, parkin deficiency can slow down PD progression in transgenic mice harboring the disease-causing mutation A30P in α-syn (Fournier et al. 2009; Lonskaya et al. 2013). Furthermore, parkin appears to contribute to Lewy body formation through K63-linked polyubiquitination of synphilin-1, a protein interacting with α-syn (Lim et al. 2005). Conversely, overexpression of synphilin-1 can suppress the neurotoxicity caused by α-syn mutation A53T (Smith et al. 2010). A study on ALS mouse models expressing mutant SOD1 demonstrated that genetic ablation of parkin delays disease progression and prolongs survival (Palomo et al. 2018). In this model, loss of parkin slows down neurodegeneration and ameliorates the loss of mitochondrial dynamics induced by ALS-related SOD1 mutant protein. A potential explanation for these unexpected effects is that the mitochondrial damage triggered by mutant SOD1 could lead to a parkin-mediated chronic activation of mitochondrial quality control, which could inhibit mitochondrial biogenesis and worsen mitochondrial dysfunction (Palomo et al. 2018). Thus, modulation of parkin might have distinct effects depending on the model organism and the proteinopathy.

UBR5 is another potentially relevant E3 ligase in the context of disease. Under normal conditions, iPSCs from HD patients do not accumulate aggregates of polyQ-expanded mutant HTT (Koyuncu et al. 2018). However, the treatment with proteasome inhibitor triggers mutant HTT aggregation in these cells, further supporting a role of the UPS in suppressing the formation of HD-related aggregates. Indeed, iPSCs express elevated amounts of UBR5 compared with their differentiated neuronal counterparts, promoting the ubiquitination of mutant HTT and its degradation by the proteasome. Notably, increasing the levels of UBR5 in HD models is sufficient to promote degradation of mutant HTT and ameliorate its aggregation (Koyuncu et al. 2018). In these lines, Yau and colleagues reported that polyQ-expanded mutant HTT is heavily ubiquitinated by heterotypic K11/K48-linked chains in cancer cells, embryonic stem cells and neurons. However, ubiquitination of mutant HTT was abolished by co-depletion of UBR5 and UBR4 (Yau et al. 2017).

Carboxy-terminus of Hsc70-interacting protein (CHIP) has the double function of E3 ligase and co-chaperone, playing an important role in UPS-mediated degradation (Ballinger et al. 1999; Jiang et al. 2001). Dysregulation of CHIP has been linked to different neurodegenerative diseases. Phosphorylation of tau is a signal for CHIP-mediated ubiquitination, and clearance of tau by CHIP increases cell survival in COS-7 cells transfected with constructs expressing both tau and CHIP (Shimura et al. 2004). In addition, CHIP knockout mice that express P301L mutant tau exhibits increased phosphorylated and caspase-3-cleaved tau accumulation (Dickey et al. 2006). Further studies demonstrated that the pathologic isoform of Aβ, namely Aβ42, decreases CHIP expression and leads to tau accumulation in 3xTg mice, which express 3 familial AD-associated mutant variants (amyloid-β precursor protein (APP) KM670/671NL, MAPT P301L, and PSEN1 M146V) (Oddo et al. 2008). A recent study on 3xTg mice reported that administrating sulforaphane, an isothiocyanate naturally found in cruciferous vegetables, increases CHIP and Hsp70 levels in mouse brains, correlating with elevated clearance of tau and phosphorylated tau. Concomitantly, sulforaphane alleviates learning and memory deficits in these mouse models, supporting an effect of CHIP upregulation at the physiological level (Lee et al. 2018a, 2018b). CHIP also ameliorates protein aggregation in distinct HD models. Zebrafish embryos injected with expanded-polyQ mutant HTT die after 24 h, but co-injection with CHIP rescues this phenotype. Moreover, deletion of a single allele of CHIP in HD mouse models hastens the aggregation of mutant HTT and the disease progression (Miller et al. 2005). A role of CHIP was also observed in ALS transgenic mouse and cell models that express mutant SOD1^G93A^. In the cell model, CHIP decreases mutant SOD1 levels by ubiquitinating Hsp/HSC70, which is an interacting partner of SOD1. In vivo proof of indirect interaction between CHIP and SOD1 was evident by co-localization of CHIP, Ub and SOD1 in the spinal cord of end-stage transgenic AD mice (Urushitani et al. 2004).

The E2 Ub conjugating enzyme UBE2K, also known as huntingtin-interacting protein 2 (HIP2), is also a potential modifier of neurodegenerative diseases. The yeast homologue of UBE2K, Ubc1, modulates aggregation of prion proteins in *Saccharomyces cerevisiae*. Deletion of Ubc1 increases prion aggregation by reducing degradation of the stress-response protein Lsb2. In turn, Lsb2 cannot promote the aggregation of prion precursor Sup35 (Chernova et al. 2011). By yeast two-hybrid system, it was discovered that human UBE2K interacts with the N-terminus of HTT protein leading to its polyubiquitination, regardless of the length of the polyQ tract (Kalchman et al. 1996). However, knockdown of UBE2K does not lead to increased levels or aggregation of mutant HTT in iPSCs from HD patients (Fatima et al. 2020). Thus, further studies are needed to assess whether UBE2K could be a modifier of HD. Besides its interaction with HTT, a potential link between UBE2K and Aβ aggregation has also been explored. In a mouse model of AD, Aβ plaques have increase UBE2K expression, which leads to stabilization of caspase-12, eventually causing neuronal death. Conversely, lowering UBE2K levels successfully induces Aβ resistance in cortical neurons and reduces activation of caspase-12 (Song et al. 2008). These results indicate that, like parkin, modulation of UBE2K could have positive or negative roles on protein aggregation depending on the disease and the models analyzed.

DUBs, the enzymes that cleave ubiquitin from proteins, are emerging as key modifiers of aging and disease. In *C. elegans*, there is an increase in global DUB activity during the aging process. Consequently, multiple proteins escape the clean-up by the UPS and accumulate with age, leading to protein aggregation and cellular dysfunction (Koyuncu et al. 2021). Moreover, deregulation of DUBs is involved in many different neurological disorders such as AD, PD, HD, and ALS (Amer-Sarsour et al. 2021; Nazé et al. 2002; Saigoh et al. 1999; Setsuie and Wada, 2007; Zeng et al. 2019). For instance, DUB activity can directly modulate the ubiquitination levels of disease-related proteins such as α-syn (Amer-Sarsour et al. 2021; Cartier et al. 2009; Guo et al. 2017; Oishi et al. 2016; Uddin et al. 2018). As such, DUBs are targets for modulating protein clearance in neurodegeneration. USP14 is a proteasome-associated DUB that can inhibit the degradation of ubiquitin-protein conjugates (Lee et al. 2010). The treatment of mouse embryonic fibroblasts (MEFs) with IU1, a small-molecule inhibitor of USP14, improves the clearance of proteotoxic tau, TDP-43, ataxin-3 (ATXN3) and GFAP, which are disease-relevant proteins in AD, ALS, Machado–Joseph disease and glia overactivation, respectively (Lee et al. 2010). Moreover, overexpression of catalytically dead USP14 reduces the levels of prion aggregates in PrP^C^-overexpressing Neuro2a mouse neuroblastoma cells (Homma et al. 2015). Importantly, knockdown of USP14 also has beneficial effects in a fruit fly model of PD, where it rescues mitophagy defects caused by PINK1/Parkin mutation and the subsequent disease-related phenotypes (Chakraborty et al. 2018). Whereas reducing USP14 activity can have beneficial effects in distinct disease models, overexpression of USP14 reduces mutant HTT aggregates and counteracts cell degeneration in neural cell lines expressing expanded-polyQ HTT constructs (Hyrskyluoto et al. 2014). In addition, USP14 does not appear to have a robust effect on the cellular levels of tau or TDP-43 in different human cell lines models such as HEK293, U2OS and SH-SY5Y (Ortuno et al. 2016). However, this might be due to methodical differences between the distinct studies (Lee et al. 2010; Ortuno et al. 2016). Nevertheless, these findings demonstrate that USP14 is a potential disease modifier, but its activity could have different effects depending on the disease or the cellular and animal models used in the assays.

USP8 is a relevant DUB in parkin-mediated mitophagy. USP8 removes K6 ubiquitination from parkin, which is required for the recruitment of parkin to mitochondria (Durcan et al. 2014). In addition to its role in mitophagy, USP8 also deubiquitinates K48 and K63-linked Ub chains on α-syn (Alexopoulou et al. 2016). Importantly, the knockdown of USP8 significantly reduces α-syn levels in SH-SY5Y human cells. This finding was further supported by a fruit fly model which ectopically expresses A53T mutant α-syn leading to a rough eye phenotype. The pathological phenotype was successfully rescued upon knockdown of USP8. However, loss of USP8 does not prevent abnormalities caused by mutant HTT (Alexopoulou et al. 2016). A further study demonstrated that USP8 can remove K11-linked polyUb chains from p62 (Peng et al. 2020), a regulator of autophagy-mediated clearance of ubiquitinated aggregates. USP8 overexpression leads to deubiquitination of p62 protein, suppressing its autophagic activity (Peng et al. 2020). However, further studies will be necessary to assess whether the impact of USP8 on autophagy influences disease-related protein aggregation. In addition to USP14 and USP8, other DUBs are also linked with neurodegenerative diseases. For instance, UCH-L1 is downregulated in patients with PD and AD (Setsuie and Wada 2007). Although the impact of UCH-L1 on disease is still enigmatic, it has been reported that UCH-L1 not only can function as a DUB, but also as an E3 ligase that extends Lys63-polyUb chains in α-syn (Liu et al. 2002). An expanded polyQ mutation in the DUB ataxin-3 causes spinocerebellar ataxia type 3 (SCA3), providing a direct link between DUBs and neurodegeneration (McLoughlin et al. 2020).

Beyond modulation of E3 or DUB enzymes, a global induction of proteasome activity can also prevent the accumulation of disease-related protein aggregates. For instance, increasing the levels of PSMD11/RPN6, a central regulator of proteasome assembly, is sufficient to increase proteasome activity (Vilchez et al. 2012a). Overexpression of *rpn-6*, the worm orthologue of PSMD11, decreases expanded-polyQ aggregation and neurotoxicity in a worm model of HD. Conversely, knockdown of *rpn-6* hastens disease-related changes, underlining the importance of PSMD11/RPN6 in removal of disease-relevant protein aggregates (Vilchez et al. 2012b). Another publication demonstrated that cAMP-mediated phosphorylation and subsequent activation of PSMD11 promotes the degradation of disease-related mutant variants of TDP-43, SOD1 and Tau (Lokireddy et al. 2015).

### Targeting UPS in cancer

Cumulative evidence demonstrates that proteasomal activity is elevated in human cancers (Arlt et al. 2009; Chen and Madura 2005; Zhang et al. 2004). Given that high proliferation rates rely on proteasome activity, proteasome upregulation is consistent with the particular requirements of malignant cells. Moreover, the elevated degree of cell divisions and mutation rates characteristic of cancer cells can lead to the accumulation of misfolded proteins, which can activate stress responses and apoptosis. Distinct studies reported protein aggregation in malignant cells (Chen et al. 2017; Chiu et al. 2019; Chou et al. 2019; Huo, 2010; Kanapathipillai 2018; Yang-Hartwich et al. 2015a,b). For instance, p53, one of the most frequently mutated proteins in human cancers, can form aggregates in cancer cells (Chen et al. 2017; Chou et al. 2019; Yang-Hartwich et al. 2015a,b). Since the proteasome eliminates aberrant or damaged proteins that otherwise would be toxic for the cell, upregulation of proteasome activity could provide an advantageous feature for cancer cells to survive amidst proteotoxic conditions (Whitesell and Lindquist 2005). Indeed, inhibition of proteasome activity is a promising therapeutic approach for the treatment of certain types of cancer (Deng et al. 2020; Devoy et al. 2005; Du and Mei 2013; Zhang et al. 2020). Although it is not clear how inhibition of the proteasome particularly affects cancer cells, a number of identified compounds that inhibit proteasome activity can induce apoptosis of malignant cells (Ling et al. 2002; Pei et al. 2003), kill tumor cells (Teicher et al. 1999), enhance radiation sensitivity (Teicher et al. 1999) and overcome drug resistance (Frankel et al. 2000; Hideshima et al. 2001). A potential explanation for this selectivity is that malignant cells show greater sensitivity to the cytotoxic effects of proteasome inhibition compared with non-cancer cells (Delic et al. 1998; Orlowski et al. 1998; Soligo et al. 2001) Bortezomib, a proteasome inhibitor that reversibly inhibits proteasome activity, is approved for the treatment of multiple myeloma (Orlowski and Kuhn 2008; Richardson et al. 2008). Multiple myeloma cells produce elevated amounts of aberrant immunoglobins and, subsequently, rely on proteasomal function for the continual clearance of abnormal proteins (Nencioni et al. 2007; Richardson et al. 2008). Bortezomib is also efficient against hematological malignancies such as Waldenström's macroglobulinemia and mantle cell lymphoma (Belch et al. 2007; Chen et al. 2007; Fisher et al. 2006; Treon et al. 2007). Two second-generation compounds have entered phase II trials; i.e., NPI-0052 and carfilzomib, which also inhibit proteasome activity but have improved pharmacological properties (Chauhan et al. 2005; Kuhn et al. 2007).

Besides global proteasome activity, other components of the ubiquitin–proteasome system can also be a potential therapeutic target for cancer. Of particular interest is UBA1, the first enzyme in the sequential ubiquitination cascade. In vitro assays with TAK-243, an inhibitor of UBA1, led to global reductions of ubiquitinated protein levels, impaired signaling, arrested cell cycle and cell death due to proteotoxic stress, which was also supported by xenograft models of cancer (Hyer et al. 2018). In 2014, the pharmaceutical company Takeda Oncology started a phase I trial with TAK-243 in patients with advanced solid tumors (NCT02045095). However, the trial was terminated due to realignment of the sponsor's pipeline program, without a publication of the existing results. Nevertheless, a new phase I trial is currently ongoing to assess TAK-243 efficiency in patients with different kinds of recurrent leukemia (NCT03816319). Distinct tripartite motif (TRIM) E3 ligases that modulate protein aggregation and proteasome activity in cancer cells are also potential therapeutic targets (Hatakeyama 2011; Meroni and Diez-Roux 2005). Among them, the E3 ligase TRIM25 is strongly upregulated under endoplasmic reticulum (ER) stress in colon and liver carcinoma cells (Liu et al. 2020a,b,c). Increased TRIM25 levels promote the removal of a transcription factor Keap1, which itself is an inhibitor of Nrf2, a regulator of antioxidant responses. In turn, Nrf2 improves survival of tumor cells under ER stress. Notably, mice grafted with stable TRIM25-knockdown cells have slower tumor progression and increased lifespan (Liu et al. 2020a,b,c). Another study revealed that TRIM11 overexpression in the colon cancer cell line HCT116 facilitates removal of both misfolded and normally folded proteins by suppressing the DUB activity of UPS14, increasing the overall proteasome activity. TRIM11 overexpression also increases cell survival after proteotoxic conditions such as heat-shock stress. When immunodeficient mice are grafted with HCT116 overexpressing TRIM11, the tumor volume expansion is significantly higher. Conversely, grafts overexpressing USP14 exhibit a slower expansion than those with endogenous levels of USP14 (Chen et al. 2018a,b). Although USP14 activity ameliorates pathological changes in this cancer model through inactivation of the proteasome, USP14 can also have pro-malignant effects in other cancer types. For instance, USP14 is upregulated in patients with lung or breast cancer. The combination of enzalutamide, a nonsteroidal antiandrogen, with either knockdown or pharmacological inhibition of USP14 promotes arrest of cell cycle progression and induces apoptosis (Xia et al. 2019). When lung cancer cells are treated either with USP14 inhibitor or USP14 siRNA, they have decreased proliferation and invasion (Han et al. 2019). Moreover, mice models with either homozygous or heterozygous deletion of p53 display slower tumor progression and increased lifespan when treated with the USP14 inhibitor IU1. In these mouse models, IU1 induces senescence, cell cycle arrest and apoptosis in malignant cells (Ma et al. 2020).

## Autophagy in disease

Deficits in autophagy are associated with multiple diseases (Frake et al. 2015; Jin and Zhang, 2020; Nixon 2013; Park et al. 2020; Towers et al. 2020; White 2015; Yin et al. 2018; Yun and Lee 2018; Zhou et al. 2019). Additionally, autophagy can be induced in many different cell types through inhibition of mTOR with different available inhibitors, most famously rapamycin (Sehgal et al. 1975). Due to its involvement in many different diseases along with the possibility of pharmacological manipulation, autophagy has been a favorable target for therapeutic approaches (Fig. 3).

### Targeting autophagy in neurodegenerative diseases

A plethora of evidence demonstrates that autophagy is involved in the clearance of aggregated proteins characteristic of neurodegenerative disorders, establishing autophagy as a central point of interest for therapeutics (Bjørkøy et al. 2005; Jung et al. 2020; Luo et al. 2020; Ravikumar et al. 2002, 2004; Sarraf et al. 2020; Webb et al. 2003). Multiple studies assessed whether induction of autophagy through rapamycin can alleviate hallmarks of AD such as protein aggregation and neuronal loss. In a mouse model for AD, which overexpresses a V717F mutant variant of human APP, rapamycin-supplemented diet improves learning and memory deficits in Morris water maze (MWM) tests. Moreover, rapamycin-treated animals have less Aβ1-42 aggregates, a clear indication of functional restoration in parallel with protein aggregation clearance (Spilman et al. 2010). An intriguing follow-up study using 3xTg-AD mice revealed that rapamycin can prevent AD only when administrated early in life, and has negligible effects on Aβ and tau aggregates when administered in advanced stages of the disease (Majumder et al. 2011). Since AD begins to develop in patients decades before the first symptoms appear, it is important to determine an administration regimen for therapeutic compounds in AD (Beason-Held et al. 2013; Lloret et al. 2019).

Recent studies on the interplay of diabetes mellitus with AD also led to promising results. Rat models of type 2 diabetes mellitus (T2DM) induced by streptozotocin (STZ) have increased dystrophic neurites together with aggregation of APP, phosphorylated tau and Aβ, mimicking AD symptoms (Li et al. 2007). However, rapamycin alleviates AD-related protein aggregation and learning deficits in these rat models through inhibition of AMPK-mTOR signaling (Sun et al. 2019). An independent study further supported these results in STZ-induced T2DM rats (Ding et al. 2021). STZ leads to hyperactivation of the mTOR/p70S6k pathway, which can be attenuated by rapamycin treatment. Rapamycin further protects against hippocampal oxidative stress damage, dysregulated mitochondrial activity, and memory impairment along with reduction of Aβ1-42 and hyperphosphorylated tau levels in the hippocampus. Despite the evidence supporting a positive correlation of autophagy induction with amelioration of AD, currently no clinical data trial data are available.

Similar to AD, cellular and animals models of HD treated with rapamycin and other mTOR inhibitors exhibit reduced protein aggregation (King et al. 2008; Ravikumar et al. 2004; Rubinsztein and Nixon 2010). A study using COS-7 cells expressing polyQ-expanded exon 1 of HTT indicates that mTOR can be sequestered into polyQ aggregates. Interestingly, sequestration of mTOR increases autophagy, as supported by increased levels of the autophagosome marker LC3-II. Moreover, the rapamycin analog CCI-779 promotes clearance of protein aggregates and ameliorates motor deficits in mice expressing mutant HTT (Ravikumar et al. 2004). A recent study demonstrated that a small molecule inhibitor of serine/threonine kinase GSK-3 can promote clearance of expanded-polyQ HTT aggregates (Rippin et al. 2021). GSK-3 was initially identified as a central kinase involved in glucose metabolism, which phosphorylates glycogen synthase, insulin receptor 1, phosphoenolpyruvate carboxykinase and glucose 6-phosphatase (Embi et al. 1980; Liberman and Eldar-Finkelman 2005; Lochhead et al. 2001). Further studies reported that inhibition of GSK-3 leads to increased autophagy in distinct cell types, mainly cancer cells (Gavilán et al. 2013; Marchand et al. 2015; Ren et al. 2018; Ryu et al. 2021). Notably, the GSK-3 inhibitor L807mts enhances clearance of aggregates through elevated autophagy in SH-SY5Y cells expressing mutant HTT. Moreover, L807mts improves motor function and coordination in R6/2 mice, a widely used mouse model for HD (Rippin et al. 2021).

Besides AD and HD, induction of autophagy via mTOR inhibition could also be a modifier of other neurodegenerative diseases. In PD models, mutant α-syn can impair autophagy itself (Xilouri et al. 2009). Rapamycin reduces death of PC12 neuronal cells treated with 6-hydroxydopamine (6-OHDA), a compound that selectively destroys dopaminergic and noradrenergic neurons mimicking the neurodegeneration characteristic of PD. Moreover, rapamycin also prevents neuronal death in mice treated with 1-methyl-4-phenyl-1,2,3,6-tetrahydropyridine (MPTP), a compound that causes selective destruction of dopaminergic neurons in substantia nigra used for PD modeling (Malagelada et al. 2010). Beyond pharmacological-induced models, rapamycin can also have beneficial effects on PD genetic models. For instance, rapamycin improves motor function in mice that express the PD-associated A53T mutant variant of α-syn, without altering the total levels of α-syn (Bai et al. 2015). A more recent study indicated that rapamycin, but not PF-4708671, a molecule that inhibits a downstream target of mTORC1 (i.e., ribosomal protein S6 kinase), can attenuate depression/anxiety-like behavior in 6-OHDA-treated mice (Masini et al. 2018). Another agent enhancing autophagy is resveratrol, which activates the AMPK/SIRT1 pathway. In a PC12 cell line overexpressing mutant α-syn, resveratrol treatment leads to increased clearance of α-syn (Wu et al. 2011). The therapeutic potential of AMPK activation is further supported by experiments in *D. melanogaster* models for PD, whereby using the agent 5-amino-1-β-d-ribofuranosyl-imidazole-4-carboxamide (AICAR) to activate AMPK reduces cell death (Ng et al. 2012). Likewise, the AMPK activating agent metformin results in decreased cell death in both MPTP-treated mice and a *D. melanogaster* model for PD (Ng et al. 2012; Patil et al. 2014). In mice overexpressing A30P mutant α-syn, the PREP-inhibitor KYP-2047 enhances the clearance of α-syn via induction of beclin-1 and subsequent enhancement of autophagy (Savolainen et al. 2014). In N2A cells overexpressing A30P and A53T α-syn, beclin-1 activation through isorhynchophylline treatment has similar effects (Lu et al. 2012). Moreover, activation of the transcription factor EB (TFEB) induces autophagy and clearance of α-syn aggregates in human neuroglioma cells overexpressing α-syn (Kilpatrick et al. 2015). An extensive discussion on autophagy-enhancing agents that ameliorate PD in various models can be found in the review published by Moors and colleagues in 2017 (Moors et al. 2017).

In addition to compounds that inhibit mTOR and induce autophagy, several studies investigated agents that can affect the lysosome (Bourdenx et al. 2016; McNeill et al. 2014; Richter et al. 2014). Mutations in *GBA1*, a gene encoding for the lysosomal enzyme β-Glucocerebrosidase (GCase), is a risk factor for PD (Sidransky et al. 2009; Sidransky and Lopez 2012). These mutations can lead to functional loss of GCase and thereby to lysosomal dysfunction and accumulation of α-syn (Bae et al. 2015; Yap et al. 2011). Ambroxol, a substrate targeting GCase, increases GCase activity and restores the lysosomal function in *GBA1* mutant fibroblasts (McNeill et al. 2014). Another substrate that targets GCase is isofagomine. Notably, the treatment with isofagomine reduces the levels of α-syn and neuroinflammation in mice overexpressing α-syn, improving their motor performance (Richter et al. 2014).

Importantly, mutations in distinct autophagy-related genes, such as *p62/SQSTM1*, *OPTN*, *C9orf72*, *ALS2*, *UBQLN2* can cause ALS (Renton et al. 2014). Indeed, defective autophagy has been reported in ALS patients and models, supporting the potential of autophagy enhancement as a therapeutic approach in ALS (Chen et al. 2018a, b; Goode et al. 2016; Lee et al. 2018a,b; Majcher et al. 2015). Nonetheless, ALS models treated with mTOR inhibitors have showed conflicting results. For instance, rapamycin does not alleviate the accumulation of protein aggregates in mutant SOD1-expresssing mice, and hastens motor neuron degeneration and organismal death in these animals (Zhang et al. 2011). A following study using a different mutant SOD1 mouse model confirmed that rapamycin do not have beneficial effects on ALS pathology, while dietary restriction increases lifespan and delays the onset of the disease (Bhattacharya et al. 2012). Nevertheless, a report using a microphysiological 3D model of ALS-related mutant TDP-43 consisting of motor neurons and muscle fibers differentiated from patient-derived iPSCs revealed that the treatment with rapamycin restores the functionality of motor neurons. Moreover, rapamycin decreases TDP-43 aggregation and apoptosis in these cells (Osaki et al. 2018). A different study using mutant OPTN-expressing mice as a model for ALS also supported the idea of rapamycin as a potential candidate for ALS treatment. Indeed, administering mutant OPTN-expressing mice with rapamycin leads to a rescue in their behavioral deficits as well as decreased TDP-43 aggregation (Zhang et al. 2021a).

Since rapamycin is a potent immunosuppressor which is also contraindicated for people with renal insufficiency, it is important to develop alternatives for autophagy induction (Baroja-Mazo et al. 2016; Ruggenenti et al. 2016). Recent advances on autophagy regulation allowed researchers to enhance autophagy through mTOR-independent pathways such as lithium administration, which is also used to treat bipolar and major depressive disorders (Motoi et al. 2014; Sarkar et al. 2005). Lithium activates autophagy pathway by inhibiting inositol phosphatase-phosphatase (IMPase). Under physiological conditions, IMPase facilitates the hydrolysis of inositol monophosphate into free inositol (Maeda and Eisenberg 1980). Inhibition of IMPase by lithium leads to depletion of free inositol, and ultimately decreases inositol triphosphate (IP3) levels (Sarkar et al. 2005). Subsequently, IP3 receptor is less active, impairing intracellular Ca^2+^-sensing mechanisms, a process that compromises mitochondrial function and ATP production. Then, increased AMP/ATP ratio activates autophagic pathways through AMPK kinase (Cárdenas et al. 2010; Decuypere et al. 2011). The treatment of AD mice models with lithium has led to mixed results. An earlier report demonstrated that the chronic treatment with lithium improves spatial learning deficiencies in rats injected with pre-formed Aβ (De Ferrari et al. 2003). Lithium also induces a significant reduction of phosphorylated tau levels in 3xTg-AD mice, but it does not has beneficial effects on Aβ aggregation or memory deficiencies (Caccamo et al. 2007). More recent studies supported that microdosing of lithium is beneficial for AD models. Mutant APP-expressing rats treated with NP03, a microdose formulation of lithium, exhibit reduced Aβ aggregation, improvements in working memory, decreased inflammation and lower oxidative stress (Wilson et al. 2020). Another study reported that APP transgenic mice treated with low doses of lithium exhibit a recovery in spatial learning. The same study found decreased levels of phosphorylated tau and Aβ aggregates in the brain of mice treated with low-dose lithium (Liu et al. 2020a,b,c). In addition, low-dose lithium treatment can also have anti-pathological effects in HD models. NP03-treated YAC128 mice, which express human expanded-polyQ mutant HTT, have improved motor function and decreased neuropathological deficits in the brain. Moreover, NP03 diminishes insoluble mutant HTT aggregates and phosphorylated tau (Pouladi et al. 2012).

In different models of PD, lithium treatment produces varying outcomes. When dopaminergic N27 cells are treated with H_2_O_2_, they have decreased survival compared to cells treated with both H_2_O_2_ and lithium, as a consequence of oxidative stress. Moreover, lithium treatment prevents oxidized/nitrated α-syn accumulation in brains of PD mice that overexpress mutant α-syn (Kim et al. 2011). In contrast, Yong and colleagues reported that 6-OHDA-induced models of PD mice do not exhibit increased survival of dopaminergic neurons upon lithium treatment. Although phosphorylated tau levels were decrease in this mouse model upon lithium, the treatment did not have any effect on PD neuropathology (Yong et al. 2011). However, another study showed enhanced dopaminergic differentiation when neural stem cells (NSCs) treated with lithium were transplanted into 6-OHDA-induced PD rat models when compared with NSCs treated with vehicle (Qi et al. 2017). In addition, NSCs treated with lithium rescue motor function in this PD model (Qi et al. 2017). Together, these data indicate that even though lithium treatment alone may not be sufficient therapy for PD, it can have complementary beneficial effects in combination with an effective treatment.

Lithium can also lead to different results depending on the ALS model. In mutant SOD1^G93A^- transgenic mice, lithium treatment prevents neurodegeneration, increases lifespan and delays the disease onset, correlating with a reduction in aggregates containing Ub and SOD1. The same study also reported a clinical trial, where ALS patients were treated with either only riluzole or riluzole combined with lithium and showed that combined therapy could have beneficial effects (Fornai et al. 2008). However, a following study refuted these claims using SOD1^G93A^-mutant mice in two different genetic backgrounds. Pizzasegola et al. (2009) found no significant differences between vehicle- or lithium-treated mice in terms of disease duration or neuroprotection. Instead, they observed an early onset of the disease and decreased survival.

### Targeting autophagy in cancer

The link between autophagy and cancer has been known since decades. However, the specific role of autophagy in cancer development remains elusive. As discussed in previous sections, mutations in the protein p53 lead to its misfolding and aggregation in cancerous cells (Carson and Lois 1995). Under physiological conditions, p53 serves as a tumor suppressor and regulates the autophagy-lysosomal pathway. Whereas nuclear p53 induces autophagy by activating the sestrin-AMPK-mTOR pathway, cytosolic p53 acts as an autophagy inactivator through mTOR (Budanov and Karin 2008; Chollat-Namy et al. 2019; Tasdemir et al. 2008). A study of Haque et al. (2018) linked cytosolic p53 aggregates with lung cancer. Using a human lung cancer cell line, they observed cytosolic p53 aggregation despite that *TP53,* the gene encoding for p53, did not harbor any mutation. The autophagic protein ATG5 co-aggregates with p53 and, subsequently, loses its physiological function. Treating lung cancer cells with the compound emodin diminishes the interaction between aggregating p53 and ATG5, leading to an increase in the autophagy flux that reduces protein aggregates. Intriguingly, induction of aggregate formation could also support anti-cancer therapies. A novel histone deacetylase inhibitor TMU-35435 can induce aggregation of misfolded proteins and thereby autophagy in triple-negative breast cancer (TNBC) (Chiu et al. 2019). Remarkably, treating mouse models of orthotopic breast cancer with both, TMU-35435 and irradiation, suppresses tumorigenesis through autophagy induction. Thus, either inhibition or activation of autophagy could have beneficial effects for cancer therapy depending on the type of cancer and the aggregated proteins.

### Targeting autophagy in inflammatory and infectious diseases

Many viruses have been reported to impinge on the autophagy-lysosome pathway. However, not all these viruses cause an infection that correlates with protein aggregation. A virus which could promote protein aggregation is the severe acute respiratory syndrome coronavirus 2 (SARS-CoV-2), which causes COVID-19. Patients with COVID-19 are predicted to have a higher risk to develop neurodegenerative diseases (Chana-Cuevas et al. 2020; Dolatshahi et al. 2021; Tavassoly et al. 2020). The analysis of potential effects of SARS-CoV-2 proteins indicates that the infection interferes with autophagosome–lysosome fusion (Miao et al. 2021; Zhang et al. 2021b). Particularly, the open reading frame 3a (ORF3a) of SARS-CoV-2 blocks the fusion between autophagosomes and lysosomes and thus the autophagic flux. ORF3a was found to interact with VPS39, a process that prevents the assembly of fusion machinery, leading to the accumulation of autophagosomes (Miao et al. 2021; Zhang et al. 2021b). Notably, disruption of ORF3a-VPS39 interaction by a point mutation diminishes the blocking effect of ORF3a. These new insights could enable researchers to develop therapies that target the fusion between the autophagosome and lysosome to diminish infection and the resulting protein aggregation. In 2019, Masaki and colleagues found protein aggregates triggered by Theiler’s murine encephalomyelitis virus (TMEV) infection. The protein TDP-43, which aggregates in ALS and FTD, exhibits abnormal cellular localization and phosphorylation upon infection with TMEV (Masaki et al. 2019).

Patients of the genetic disorder cystic fibrosis (CF) typically present chronic inflammation in their lungs. Interestingly, CF patients display the accumulation of protein aggregates in their airways hinting to a possible role of protein clearance mechanisms such as autophagy (Brockman et al. 2017; Luciani et al. 2010). CF is caused by mutations in the cystic fibrosis transmembrane conductance regulator (CFTR) (Ratjen and Döring 2003). In 2010, Luciani and colleagues linked defective CFTR with dysfunctional autophagy and reduced clearance of aggresomes (Luciani et al. 2010). Since then, many studies sought to understand the link between CFTR and autophagy to develop therapeutic approaches. Targeting macroautophagy by silencing BAG3, a co-chaperone that mediates selective macroautophagy, corrects trafficking defects caused by the disease-related F508del-CFTR mutant variant. A similar effect was reported for other disease-causing mutations in CFTR, i.e., G85E, R560T and N1303K. Although targeting the UPS by silencing BAG1 also has beneficial effects, targeting autophagy provides more promising results (Hutt et al. 2018).

## Clinical trials on proteolytic systems to prevent protein aggregation

One of the challenges for the clinical treatment of proteinopathies is the selection of targets and drugs. The UPS, autophagy, and the aggregating proteins themselves might be a potential target for disease intervention (please see Table 2 for a summary of clinical trials and preclinical studies discussed in this section). Furthermore, the rationale on how a drug would affect the course of the disease is also different for cancer and neurodegeneration. Regarding cancer, the primary goal is to prevent protein clearance mechanisms from functioning properly with the aim to induce a proteostasis collapse in malignant cells, leading to reduced proliferation and invasion (Almond and Cohen, 2002; Crawford et al. 2011; Liu et al. 2020a,b,c; Manasanch and Orlowski 2017; Mulcahy Levy et al. 2017; Mulcahy Levy and Thorburn 2020). In neurodegenerative diseases that involve protein inclusions, many approaches seek to upregulate or rescue protein clearance systems to prevent pathological protein aggregation (Corti et al. 2020; Menzies et al. 2017; Nah et al. 2015; Schmidt et al. 2021; Watanabe et al. 2020). As ongoing therapies and clinical trials for cancer have been discussed in previous sections, here we will focus on interventions that could alleviate neurodegenerative diseases. The disparities between the pathophysiology of distinct proteinopathies led to exploring many different types of interventions for preventing protein aggregation (Arosio et al. 2014; Hyun and Shin 2021; Lashuel 2021; Salahuddin et al. 2021). Such interventions can be classified as antibodies, protein stabilizers, nanoparticles, sequestering monomers and small molecule inhibitors of aggregation.

**Table 2:** List pre-clinical and clinical trials to prevent protein aggregation and ameliorate neurodegenerative diseases

Disease	Agent	Effect	Clinical Phase	Trials	Publication
AD	Immunization against Aβ42	Reduced neuronal Aβ-plaque deposition; ameliorates behavioral deficits	Pre-Clinical	–/–	Schenk et al. (1999)
AD	Curcumin	Prevent aggregation of tau, Aβ and α-syn	Pre-Clinical	–/–	Pandey et al. (2008), Rane et al. (2017), Sharma and Nehru (2018), Yang et al. (2005)
AD	Aducanumab	Monoclonal antibody targeting Aβ	Approved	NCT02484547	Dunn et al. (2021)
AD	Tafamidis meglumine	Prevents amyloidogenesis	Approved	–/–	Unpublished
AD	sulforaphane	–/–	Recruiting	NCT04213391	Unpublished
AD	Rapamune	–/–	Early phase 1	NCT04200911	Unpublished
AD	Trehalose	–/–	Phase 1	NCT04663854	Unpublished
AD	Curcumin	No clinical or biochemical improvements	Phase 2	NCT00099710	Ringman et al. (2012)
AD	Epigallocatechin-Gallate	Prevents the aggregation of beta-amyloid	Phase 2	NCT00951834	Unpublished
AD	Lithium Carbonate	Mitigated cognitive decline; modified AD-related CSF biomarkers	Phase 2	NCT01055392	Forlenza et al. (2019)
AD	Methylene blue; TRx0014	Improvement of cognitive function	Phase 2	NCT00515333	Wischik et al. (2015)
AD	Rapamycin	–/–	Phase 2	NCT04629495	Unpublished
AD	5-HT6 antagonist; SB-742457	Improvement of cognitive function	Phase 2	NCT00348192; NCT00710684; NCT00708552	Maher-Edwards et al. (2015, 2010)
AD	Hydralazine hydrochloride	–/–	Phase 3	NCT04842552	Unpublished
AD	Leucomethylene blue; TRx0237	–/–	Phase 3	NCT03446001	
AD	Leucomethylene blue; TRx0237	Reduced brain atrophy	Phase 3	NCT01689246	Wilcock et al. (2018)
ALS	Colchicine	–/–	Phase 2	NCT03693781	Cadwell (2016), Mandrioli et al. (2019), Zhao et al. (2015)
ALS	Rapamycin	–/–	Phase 2	NCT03359538	Unpublished
ALS	Tamoxifen	Moderate effects on ALS score of functional scale	Phase 2	NCT02166944	Chen et al. (2020)
PD	Nilotinib	Improved cognitive and motor functions	Early Phase 1	NCT02281474	Pagan et al. (2016)
PD	Ambroxol	Improvement of cognitive function	Phase 2	NCT02914366; NCT04388969	Mullin et al. (2020)
PD	Nilotinib	No symptomatic benefits	Phase 2	NCT03205488	Simuni et al. (2021)

The disease, molecular agent and its effects as well as the clinical trial status are indicated. Trial numbers derived from clinicaltrials.gov

Immunization with antibodies against toxic protein aggregates provided promising results in preclinical studies. An early study demonstrated that immunization against Aβ_42_ reduces neuronal Aβ-plaque deposition and ameliorates behavioral deficits in AD mouse models that overexpress human APP (Schenk et al. 1999). Two further independent studies supported these findings, boosting the confidence in immunization studies (Janus et al. 2000; Morgan et al. 2000). A potential explanation for these beneficial effects is that antibodies promote the clearance of amyloid plaques through phagocytosis mediated by Fc receptor, which is a surface protein found in many different types of immune cells (Bard et al. 2003; Salahuddin et al. 2021). Unfortunately, these studies came to a halt when early clinical trials resulted in serious side effects, including a death due to meningoencephalitis (NCT00021723) (Neugroschl and Sano 2010). In June 2021, the United States Food and Drug Administration (FDA) approved aducanumab, a monoclonal antibody targeting Aβ in brains of patients in early stages of AD, providing a new hope for immunization against proteinopathies (Dunn et al. 2021). A similar immunization strategy was also investigated for aggregated SOD1 in ALS. Active immunization against human SOD1^G93A^ induced the clearance of SOD1 in the spinal cord of mice expressing SOD1^G37R^ and extended their lifespan by more than 4 weeks. However, it only conferred protection for SOD1^G93A^- expressing mice by passive immunization, but not by active immunization (Urushitani et al. 2007). Different studies for developing a vaccine using either wild-type SOD1 or disease-related variants of SOD1 showed extension in lifespan and delay in disease progression (Takeuchi et al. 2010; Zhao et al. 2019). Yet, no clinical trials have been conducted for SOD1 immunization.

Protein stabilizers can be described as other proteins, peptides or small molecules that bind to a protein and prevent it from unfolding or aggregation. For instance, phthalocyanine tetrasulfonate (PcTs) can interact with the N-terminal region of α-syn, leading to its stabilization through salt bridges and π–π stacking interactions (Bisi et al. 2021; Lee et al. 2004). Importantly, PcTs can reduce cell death, fibril formation and amyloidosis induced by wild-type or mutant α-syn (Fonseca-Ornelas et al. 2014; Lamberto et al. 2009; Lee et al. 2004). Although PcTs also appear to prevent the formation of aggregates from PrP, Aβ and tau, it has not been investigated in clinical trials (Valiente-Gabioud et al. 2016). Tafamidis meglumine is a successful protein stabilizer that has been translated into a drug for disease intervention. Particularly, it was developed as a stabilizer for transthyretin (TTR), a serum transport protein. Under physiological conditions, TTR transports thyroid hormone T4 and retinol bound to retinol-binding protein as a tetramer complex. Certain mutations destabilize TTR tetramers and leads to its amyloidogenesis, which can cause rare diseases such as amyloid cardiomyopathy, senile systemic amyloidosis and amyloid polyneuropathy (Ruberg and Berk 2012). Tafamidis meglumine prevents TTR tetramer dissociation and the subsequent amyloidogenesis (Connelly et al. 2010). After successfully passing phase II and III of clinical trials, it was approved by FDA for treatment of TTR amyloidosis.

Regarding nanoparticles, this approach has several advantages over small molecules such as their ability to pass through blood–brain barrier (BBB) as well as their flexibility in size, charge and release rate of their cargo (Mudshinge et al. 2011; Patra et al. 2018). Many nanoparticles with different loads have been investigated for modifying distinct disease-related changes, such as mitochondrial dysfunction, inflammation and excitotoxicity, but here we will focus on nanoparticles and nanobodies targeting protein aggregation (Baskin et al. 2021; Mushtaq et al. 2015; Wang et al. 2020a,b). An example is epigallocatechin gallate (ECGC), a polyphenol which reduces the formation of α-syn aggregates in cell-free environments, in vitro cultured neurons and animal models (Bieschke et al. 2010; Caruana et al. 2011; Kurnik et al. 2018; Xu et al. 2017). However, ECGC fell short of a successful clinical trial because its high hepatotoxicity and inefficacy in patients with multiple system atrophy (NCT02008721) (Levin et al. 2019). This could be partly attributed to poor blood–brain barrier penetration of ECGC, and inefficient uptake by dopaminergic neurons (Baskin et al. 2021). Li et al. addressed this issue using self-assembled B6 nanoparticles, which are peptides with a high affinity for transferrin receptor, coated with ECGC and mazindol, a drug with high affinity for dopamine transporter. Nanoparticle-delivered ECGC successfully accumulated in substantia nigra, and improved behavioral deficits and biomarkers in a mouse model of PD when compared with free ECGC (Li et al. 2018). In addition to PD, nanoparticles were also investigated as a potential approach to modify disease-related protein aggregation in AD. In a recent study, Zhang and colleagues described IS@NP/KH, a bifunctional nanoparticle made of chitosan which is coated with both an Aβ oligomer-binding peptide and a brain-targeting peptide. Nasal administration of IS@NP/KH to APP/PS1 mice, which express both mutant APP and presenilin, attenuates cognitive decline, improved motor function and decreased amyloid plaques in the brain (Zhang et al. 2021c). In addition to multiple system atrophy, ECGC has also been investigated as a treatment for different aggregation pathologies. A phase II trial using ECGC for treating light-chain amyloidosis failed to demonstrate the efficacy of ECGC on improving the prognosis of the disease (Meshitsuka et al. 2017). Furthermore, two different phase II trials using ECGC for HD (NCT01357681) and AD (NCT00951834) have been completed, but their results are yet to be published.

Among small molecules, curcumin is a potential treatment to ameliorate protein aggregation. Curcumin is a natural phenol which can prevent aggregation of tau, Aβ and α-syn in cell-free conditions, both in vitro and in vivo (Pandey et al. 2008; Rane et al. 2017; Sharma and Nehru 2018; Yang et al. 2005). Unfortunately, the preclinical success of curcumin did not warrant positive clinical outcomes. A phase II study on AD patients showed no clinical or biochemical improvements of subjects after 24 weeks of curcumin administration (NCT00099710) (Ringman et al. 2012). Although the potential effects of curcumin on other proteinopathies are yet to be explored in a clinical context, it presents a challenge due to its inadequate efficacy ensued from low bioavailability (Anand et al. 2007). Methylene blue (MB) and leucomethylene blue (LMTM) has been long known to inhibit the formation of tau and α-syn aggregates. As such, these compounds can improve behavioral deficits in mice models of tau and α-syn aggregation (Masuda et al. 2006; Melis et al. 2015; Schwab et al. 2018; Taniguchi et al. 2005; Wischik et al. 1996). A phase II study using MB for treating mild or moderate AD showed the potential of MB to improve cognitive function in AD patients after 24 weeks of treatment (NCT00515333) (Wischik et al. 2015). A phase III study demonstrated that LMTM can reduce brain atrophy in patients after 9 months of drug administration (NCT01689246) (Wilcock et al. 2018). As of August 2021, another phase III trial is being conducted to assess the safety and efficacy of LMTM on AD patients (NCT03446001).

Due to a lack of availability of compounds that can activate the proteasome machinery, interventions to modulate autophagy through small molecules are in more advanced phases. For instance, 5-hydroxytryptamine receptor 6 (5-HT6) antagonists have gained attention as potential candidates for AD treatment. Interestingly, 5-HT6 antagonists increase mTOR activity, and theoretically should suppress autophagy (Meffre et al. 2012). Phase II trials (NCT00348192; NCT00710684; NCT00708552) using a 5-HT6 antagonist, SB-742457, only showed slight improvements in cognition of AD patients (Maher-Edwards et al. 2010, 2015). Likewise, lithium could not induce changes in the concentration of cerebrospinal fluid (CSF)-derived biomarkers and did not improve cognitive functions of AD patients in a 10-week treatment regimen (ISRCTN72046462) (Hampel et al. 2009). However, a 2-year treatment with lithium of patients with amnestic mild cognitive impairment (NCT01055392) mitigated their cognitive decline and also modified AD-related CSF biomarkers (Forlenza et al. 2019). Nonetheless, autophagy induction for AD treatment is still a promising approach. In these lines, different clinical studies using rapamycin (NCT04629495; NCT04200911), trehalose (NCT04663854) and hydralazine (NCT04842552) are currently active with some of them still being in the recruitment stage.

Nilotinib, an inhibitor of tyrosine kinase Abelson (c-Abl), was tested as a disease-modifying compound due to its enhancing effects on autophagy through activation of the kinase AMPK (Hussain et al. 2019; Karim et al. 2020; Karuppagounder et al. 2014; Yu et al. 2013). Particularly, inhibition of c-Abl via Nilotinib may have beneficial effects on PD patients. Induction of c-Abl can lead to the phosphorylation and inhibition of parkin E3 ligase activity, resulting in the accumulation of Parkin Interacting Substrate (PARIS) (Shin et al. 2011). The toxic increase of PARIS subsequently leads to mitochondrial dysfunction and loss of dopaminergic neurons (Shin et al. 2011). An early phase I trial of Nilotinib (NCT02281474) with 12 patients presented favorable but transient effects on PD patients. In this trial, patients were treated with either 150 mg or 300 mg of Nilotinib daily for 24 weeks. Whereas cognitive and motor functions improved transiently, they deteriorated again for both groups once the treatment was discontinued. In addition, the treatment group had serious adverse events such as urinary tract infection, pneumonia, myocardial infarct and psychotic symptoms which required further studies to clarify the potential therapeutic effects of Nilotinib (Pagan et al. 2016). A phase II trial with 76 participants (NCT03205488) revealed that Nilotinib did not provide any symptomatic benefits to PD patients. Furthermore, they confirmed that the penetrance of the drug in the CSF was low and that the levels of dopamine metabolites were unchanged (Simuni et al. 2021).

The medication Ambroxol is also a potential therapeutic approach for PD. Since Ambroxol has mucolytic activity, it is typically used as a pharmacological chaperone for airway diseases (Su et al. 2004). Interestingly, Ambroxol indirectly enhances autophagy by raising the levels of GCase which in turn decreases the levels of α-syn. Treatment with Ambroxol results in increased LC3-II levels and lysosomal content (Choi et al. 2018; Magalhaes et al. 2018; Moors et al. 2017). A phase II trial on a small cohort of 17 PD patients demonstrated that Ambroxol treatment for 6 months improved their cognitive functions. Although the results are promising, this was a non-randomized, non-controlled trial which requires further investigation (Mullin et al. 2020). Two additional studies aiming to elucidate the effects of Ambroxol on PD pathology are in the recruitment stage (NCT02914366; NCT04388969).

To enhance protein clearance mechanisms in ALS, Chen and colleagues recently used Tamoxifen in a phase II critical trial (NCT02166944) (Chen et al. 2020). Tamoxifen is an anti-cancer drug that binds to estrogen receptor and inhibits cancer cell growth (Goodsell 2002; Shiau et al. 1998). Tamoxifen can also upregulate autophagy through both mTOR-dependent and -independent pathways (Cho et al. 2012; Kaverina et al. 2018; Torres-López et al. 2019). In their randomized double-blind trial, Chen et al. could only detect modest and transient effects of Tamoxifen treatment for 12 months. The decline in score of functional scale for ALS (ALSFRS-R) was slower in Tamoxifen-treated group for the first 6 months, but after 12 months the ALSFRS-R scores were identical between placebo and Tamoxifen-treated groups (Chen et al. 2020). Mandrioli and colleagues are currently recruiting patients with sporadic ALS for a phase II clinical trial (NCT03693781) using Colchicine, an anti-inflammatory drug. Besides its anti-inflammatory effects, Colchicine increases the mRNA and protein levels of the heat shock protein B8 (HSPB8). HSPB8 is a component of the chaperone-assisted selective autophagy machinery that promotes the removal of ALS-related variants of SOD1 and TDP-43 as well as aggregation-prone dipeptides derived from mutant C9orf72 (Crippa et al. 2016; Cristofani et al. 2017, 2018). Similar to other neurodegenerative diseases, the activation of inflammasome complexes in microglia and astrocytes in response to protein aggregation causes neuroinflammation that contributes to the neurodegeneration characteristic of ALS (Cadwell 2016; Mandrioli et al. 2019; Zhao et al. 2015). Since autophagy can downregulate the inflammasome activity triggered by aggregation of disease-related proteins, compounds such as Colchicine that target both neuroinflammation and autophagy are a promising approach for ALS treatment (Cadwell 2016; Mandrioli et al. 2019; Zhao et al. 2015). Another phase II study (NCT03359538) started in 2017 is assessing the effects of rapamycin-induced autophagy on ALS patients. Recently, this clinical trial completed primary data collection. However, the results are not yet publicly available.

Clinical data using molecules targeting UPS as a treatment for proteinopathies are much more limited. As mentioned above, IU1, an inhibitor of DUB activity of USP14, was reported to increase proteasomal degradation of several disease-related proteins (Lee et al. 2010). However, there are no clinical trials investigating the efficacy of IU1 for neurodegenerative diseases. On the other hand, a proteasomal enhancer sulforaphane is in early clinical phases. Notably, sulforaphane enhances the three proteolytic activities of the proteasome, i.e., chymotrypsin-like, caspase-like and trypsin-like activities in the brains of mice (Liu et al. 2014). Currently, a clinical trial conducted by Zhejiang University is in the recruitment phase for AD patients to assess the therapeutic potency of sulforaphane (NCT04213391).

## Conclusions

Proteinopathies are complex, multifaceted diseases that lead to or exacerbate neurodegenerative and immune system disorders as well as cancer (Grimaldi et al. 2018; Kanapathipillai 2018; Kumar et al. 2016; Yang-Hartwich et al. 2015a,b). Although the clearance of damaged and aggregated proteins proves to ameliorate neurodegenerative disease-related changes in cellular and organismal models, a complete understanding of their regulatory mechanisms and how they can be modified to prevent disease is far from understood. Having discrete models of such proteinopathies is a first step, but researchers bear in mind that a single model cannot encapsulate all the aspects of a disease. This is evident by distinct models of neurodegenerative diseases responding differently to the same drug regimens (Fornai et al. 2008; Kim et al. 2011; Pizzasegola et al. 2009; Yong et al. 2011). Further combined endeavors from basic science and translational approaches will continue elucidating potential mechanisms for treating proteinopathies.
